# Achieving Cultural Congruency in Weight Loss Interventions: Can a Spirituality-Based Program Attract and Retain an Inner-City Community Sample?

**DOI:** 10.1155/2014/641939

**Published:** 2014-04-07

**Authors:** Chad Davis, William Blake Dutton, Taryn Durant, Rachel A. Annunziato, David Marcotte

**Affiliations:** Department of Psychology, Fordham University, 441 E. Fordham Road, Bronx, NY 10458, USA

## Abstract

Ethnic minorities continue to be disproportionately affected by obesity and are less likely to access healthcare than Caucasians. It is therefore imperative that researchers develop novel methods that will attract these difficult-to-reach groups. The purpose of the present study is to describe characteristics of an urban community sample attracted to a spiritually based, weight loss intervention. * Methods.* Thirteen participants enrolled in a pilot version of Spiritual Self-Schema Therapy (3S) applied to disordered eating behavior and obesity. Treatment consisted of 12 one-hour sessions in a group therapy format. At baseline, participants were measured for height and weight and completed a battery of self-report measures. * Results.* The sample was predominantly African-American and Hispanic and a large percentage of the sample was male. Mean baseline scores of the EDE-Q, YFAS, and the CES-D revealed clinically meaningful levels of eating disordered pathology and depression, respectively. The overall attrition rate was quite low for interventions targeting obesity. * Discussion.* This application of a spiritually centered intervention seemed to attract and retain a predominantly African-American and Hispanic sample. By incorporating a culturally congruent focus, this approach may have been acceptable to individuals who are traditionally more difficult to reach.

## 1. Introduction

Obesity has vast, extensively reviewed health consequences [1] such as diabetes, hypertension, dyslipidemia, retinopathy, neuropathy, and cardiovascular disease [2]. The obesity epidemic has spawned thousands of research initiatives over the years but few have resulted in long-term weight loss [3, 4]. When compared to Caucasians, ethnic minorities in this country continue to disproportionately suffer from obesity. Among adults, roughly 41% of African-Americans and over 38% of Hispanic-Americans are considered obese, compared to less than 33% of non-Hispanic White Americans [5]. Researchers are therefore charged with developing and testing novel weight loss interventions that will both attract and retain Hispanic-American and African-American participants.

Compared to Caucasians, Hispanic-Americans and African-Americans are less likely to access health care services [6–9], which may make it more difficult to enroll ethnically diverse samples from clinical settings. African-Americans are also less likely to seek professional assistance for weight loss [10], as well as to trust and feel respect from healthcare providers [11]. As a result, African-American women, in particular, have expressed a preference for managing medical and mental health issues “on their own” [12], which suggests that a community-based approach to weight control may be more acceptable to this population and potentially more effective than hospital-based programs.

## 2. Spirituality and Therapy

Using religion as a coping mechanism has been shown to be a protective factor against stressful life events while weekly religious attendance has been associated with improvements in depressive symptoms [13]. According to the Gallup Poll [14], most Americans surveyed believe in God and associate with a religion. Thus, it is not surprising that many patients talk about their spiritual or religious beliefs in medical and mental health settings [15]. The American Association of Pastoral Counselors and The Samaritan Institute [16] surveyed one thousand Americans and found that 83% of the participants believed that their spiritual beliefs were tied to their mental health and 75% preferred therapists who integrated spirituality into treatment. Stanley et al. [15] surveyed 66 adults over the age of 55 who had received therapy for depression and anxiety with a comorbid medical illness. The researchers found that over 77% of the participants wanted their spiritual beliefs to be incorporated into treatment. In addition, the authors proposed that the integration of spirituality into therapy would help to attract inner-city minorities and people in rural settings who usually seek out help from their religious leaders. Many adults turn to religious leaders for counseling; however, issues that require psychotherapy are often not adequately addressed within faith-based organizations [17].

Religious commitment has been found to be associated with higher rates of obesity [18]. In a study that examined obesity rates across religious denominations, Conservative Protestant men were found to be heavier than men who did not affiliate with a religion [19]. Interestingly, Dodor [20] found that African-Americans who prayed often and deemed their religion to be important to them had poor dieting habits. Theories that have attempted to explain causal relationships between religious commitment and obesity have not yet been uniformly supported but one thing seems clear: obesity is prevalent in religious communities and developing weight loss interventions that will attract spiritually minded individuals seems to be a logical next step in addressing the obesity epidemic, perhaps especially among ethnic minorities.

## 3. The Present Study

The goal of the present paper is to describe characteristics of an urban community sample attracted to a spiritually based weight loss intervention, outline strategies used to recruit the sample, and show that this type of study was successful in retaining a difficult-to-reach population. We hypothesized that ethnic minorities from the community would enroll in a spiritually focused intervention and complete the majority of the group therapy sessions.

## 4. Methods

### 4.1. Approach

Spiritual Self-Schema Therapy (3S) is a manual-guided, empirically validated treatment originally designed to increase motivation for drug abstinence in HIV positive, injection drug abusers [21]. It uses a nontheistic approach to increase accessibility, cognitive-behavioral techniques, Buddhist psychology, and the individual's personal spirituality to increase motivation for treatment. The program is constructed to engage the emotional and motivational processes of change [21]. This feature of the intervention reflects its potential for addressing maladaptive behavior in multiple domains, including eating pathology. The intervention teaches specific behavioral skills to increase mindfulness and reduce harmful behavior in 12 one-hour group sessions (see Table 1 for an outline of topics covered in each weekly session). Brief assessments were given before and after the intervention. Treatment sessions were delivered by a doctoral-level clinical psychologist and the study was approved by the Institutional Review Board at Fordham University.

### 4.2. Intervention Development

The senior author was one of the original developers of Spiritual Self-Schema Therapy (3S) [22] and hypothesized that it might be useful if applied to populations who engaged in overeating. In collaboration with the other investigators, he subsequently reviewed the extant obesity literature and consulted with experts in the obesity field. The Food Addiction (FA) model was considered because of its strong consistency with the 3S framework. However, we acknowledge that support for the FA model is equivocal. To our knowledge, there are no published studies that have tailored weight loss interventions directly to the construct of food addiction so it is unclear if such an attempt would be superior or inferior to other types of interventions in reducing weight and eating disordered behaviors.

Culturally sensitive treatments for other types of addiction have been implemented in a variety of settings, including community sites, and have demonstrated effectiveness in helping African-Americans abstain from smoking [23] and the abuse of illegal substances [24]. It is unclear at this time if ethnic minorities identify with the construct of food addiction and are likely to enroll in a study that frames eating pathology in this way but an intervention that incorporates spirituality may draw in such participants. Given these considerations, the investigators decided to go forward with a pilot of 3S to see if the original manual could be applied to an obese population without any modifications (except for replacing references to “drugs” with “addictive eating behaviors”).

### 4.3. Recruitment

Two online advertisements describing a spirituality-focused weight loss program were broadcast within one week of each other. There were 112 responses to the advertisements. Study staff responded to all emails, informing interested parties that the first twenty participants to confirm availability would be invited to attend a free, one-hour orientation session that described the treatment. Twenty participants (*N* = 20) attended the orientation and thirteen (*N* = 13) participants returned the following week to give informed consent, complete the preassessment battery, and participate in the first session of the therapy. All participants were compensated with $10 in cash for each group therapy session attended. All sessions were held in a conference room located in the Center for Community Engaged Research (CCER) at Fordham University.

### 4.4. Measures

The Yale Food Addiction Scale (YFAS) has its roots in addictive behavior modification. It is a 27-item, self-report measure that uses Likert and “Yes/No” questions to detect the presence of food addiction, as conceptualized by the original authors of the scale. The scale's questions were originally inspired by Substance Dependence criteria outlined in the DSM-IV-TR [25]. The scale has been shown to have adequate validity and reliability [25]. A continuous mean score, ranging from endorsement of 0 to 7 symptoms, can be calculated.

The Eating Disorder Examination-Self-Report Questionnaire (EDE-Q) is a gold-standard instrument used to detect eating disordered behaviors across a wide variety of samples [26]. The EDE-Q can also be used as a proxy for diagnosing eating disorders (Anorexia Nervosa, Bulimia Nervosa, Binge Eating Disorder, and ED-NOS). The EDE-Q consists of 41 items based on questions from the Eating Disorder Examination, a structured clinical interview used in research settings [27]. The EDE-Q produces an overall Global score and consists of four subscales: Restraint, Weight Concern, Shape Concern, and Eating Concern. The EDE-Q and its subscales have been found to have adequate validity and internal consistency [28].

In a recent study conducted by Aardoom et al. [29] new EDE-Q norms were generated comparing healthy controls, obese participants, and participants with eating disorders. Healthy volunteers had a mean EDE-Q Global score of 0.93 (SD = 0.86), participants with eating disorders had a mean score of 4.02 (SD = 1.28), and obese participants had a mean score of 2.75 (SD = 0.97).

The Center for Epidemiological Studies Depression Scale (CES-D) is a widely used self-report instrument purported to measure depressive symptoms [30]. The CES-D has been shown to have acceptable validity, test-retest reliability, and high internal consistency [30]. The CES-D gives scores between 0 and 60, with higher scores being indicative of distress. Participants who score greater than 16 on the CES-D may be experiencing clinically significant psychological distress.

The Brief Multidimensional Measure of Religiousness/Spirituality (BMMRS) is a 40-item self-report scale, specifically developed for health researchers to examine different aspects of spirituality as they relate to mental and physical health outcomes [31]. The present study focused on two of the scale's dimensions: Daily Spiritual Experiences and Religious/Spiritual Coping. Daily Spiritual Experiences is a 6-item domain that intends to measure how spirituality impacts the individual's day-to-day life (e.g., a sense of connection with God). Using a 6-point Likert scale, participants rate the extent to which they perceive a daily relationship with a greater power (with “1” being low and “6” being high). Religious/Spiritual Coping is a 7-item subscale that intends to measure how an individual uses spirituality to cope with stressors and answer life's big questions. Using a 4-point Likert scale, participants rate the extent to which spiritual practices are employed in their lives (with “1” being low and “4” being high). Because the subscale includes both positive and negative aspects of religious coping, we reverse-scored the negative factors in our analysis, as recommended. The BMMRS and its domains have demonstrated adequate validity and reliability [31, 32].

Self-report questionnaires were given to assess gender, racial identification, income level, employment status, and marital status. A hospital scale was used to measure participants' weight and height at baseline and body mass index (BMI) was calculated from these data.

### 4.5. Statistical Plan

Data were analyzed using SPSS version 19.0 (SPSS Inc., Chicago, IL, USA) for Windows. Descriptive statistics were generated to analyze characteristics of the sample at baseline.

## 5. Results

### 5.1. Community-Based Recruitment

Initial recruitment efforts employed a Community-Based Participatory Research (CBPR) design that attempted to establish partnerships with religious organizations in Harlem and The Bronx, NY. However, a year of petitioning was unsuccessful in generating interest from religious groups. No religious leaders responded to inquiries from the study staff so it was impossible to learn the reasons behind the unresponsiveness. Past literature has identified challenges to getting community-based projects off the ground. Difficulties include lack of community interest in the funded topic, discrepancies between academic and community approaches to conducting research, the time, organizational structure and funding required, and mistrust of researchers by members of the community [33, 34]. It is possible that this last issue, mistrust in researchers, may be particularly germane to the initial, unsuccessful recruitment efforts of the current study. African-Americans have historically been taken advantage of in research [35] and invitations from unfamiliar academics may have been understandably dismissed.

Initial recruitment efforts for a preintervention focus group on “weight loss” were also unsuccessful. After posting 2 online advertisements, only 3 people attended the focus group. Conversely, an online advertisement that read “Join a Spiritually-Focused Weight-Loss Study” resulted in an overwhelming response (*N* = 112).

### 5.2. Characteristics of the Sample

The mean age for the total sample (*N* = 13) was 42.46 years (SD = 9.54). Males made up 38.5% (*N* = 5) of our participants. In the baseline sample, 38.5% were identified as African-American (*N* = 5), 15.4% Caucasian (*N* = 2), 15.4% Multiracial (*N* = 2), and 30.8% Hispanic (*N* = 4). Fifty-four percent (*N* = 7) of the sample were identified as single and 23.1% (*N* = 3) had a high-school education or less. Sixty-one percent (*N* = 8) reported making less than $30,000 a year. Only 15.4% (*N* = 2) had full-time employment at the beginning of the study. A summary of sample characteristics can be found in Table 2.

The mean BMI was 36.2 (SD = 4.75). All participants had a BMI > 30, which places them all in the obese range. Participants reported a mean YFAS score of 4.00 (SD = 2.31) indicating that they endorsed an average of 4 out of 7 symptoms. The mean EDE-Q Global score was 3.27 (SD = 1.53). Compared to the norms outlined in past literature [29, 36], it appears that the eating pathology of this sample is more severe than the average obese person and substantially more severe than the general population.

The mean score on the CES-D was 23.85 (SD = 10.67), indicating clinically significant levels of depression. Furthermore, 61.5% of the sample had scores greater than 16, which suggests that the vast majority was suffering from clinically significant psychological distress at baseline.

The mean score on the BMMRS Daily Spiritual Experiences Domain was 4.71 (SD = 1.16) and the mean score on the BMMRS Religious Coping Domain was 3.19 (SD = 0.57). Given that the Daily Experience Domain uses a 6-point Likert scale and the Religious Coping Domain uses a 4-point scale, the means and standard deviations suggest that the majority of our participants often pray, use faith, or meditate to help them cope with life's stressors and have relationships with a higher power or experience the presence of spiritual entities in their daily lives.

### 5.3. Retention and Attendance

Participants who completed 10 of the 12 group therapy sessions were defined as “completers.” Past psychotherapy studies have used similar attendance criteria when defining completers [37, 38]. Interestingly, the only 2 participants who were identified as Caucasian dropped out. One participant stopped coming after attending session 4 and the other after attending session 6. One of these participants mentioned from the outset that she frequently worked late but was trying to adjust her schedule so she could attend the sessions. It is unknown why the other participant dropped out. In total, 11 participants, all of whom were ethnic minorities, attended 94.5% of all sessions, on average, and 8 of those participants completed all the 12 sessions. Several of the participants asked if they could bring a friend or family member and nearly all stated that they would recommend the treatment program to a friend.

## 6. Discussion

Although limited by modest recruitment goals, a hard-to-reach sample was successfully recruited and retained in a 12-session treatment that utilized personal spirituality as a component of the intervention. A balanced group of male and female, obese minorities from inner-city communities in Harlem and The Bronx responded enthusiastically to internet-based recruitment efforts and participated eagerly in all group sessions.

Advertising a weight loss intervention that incorporated spirituality attracted a predominantly Hispanic-American and African-American sample with strong spiritual/religious beliefs. Boltri et al. [39] conducted a focus group to identify barriers to engagement with a church-based diabetes mellitus prevention program for African-Americans. Qualitative analyses revealed that spirituality was an integral part of how participants conceptualized their own health and related behaviors and prayer was integral to how several participants coped with the challenges of illness [39]. These findings may suggest at least one reason why the use of spirituality in the current study was highly attractive to a predominantly African-American sample. Simply, spirituality matters and motivation for spirituality-related goals is high in this group. At the least, this suggests that incorporating spiritual dimensions into treatment for obesity may increase adherence in cohorts that are traditionally difficult to engage. It is however unknown if a spiritually centered intervention such as ours will result in meaningful changes in weight and reductions in eating disordered symptoms.

Although the idea of “food addiction” is relatively new and largely untested with obese populations, this sample readily accepted the proposal that their weight problems could be related to addictive eating behaviors and agreed without objection that being “addicted to eating” accurately reflected their own experience. Relating obesity to addictive behavior may provide a way to conceptualize and treat traditionally resistant patterns by focusing on discrete eating behaviors that can be identified and changed one at a time. Recognizing an aspect of addictive behavior in their eating patterns can also help individuals to identify cognitive and affective precursors that may be propitious points of intervention. Qualitative data may illuminate whether and how individuals identify with food addiction as a part of their illness.

Albeit small, this sample was almost 40% male. This is unusual in obesity studies where men tend to be largely underrepresented [40]. Since weight loss is traditionally associated with the attainment of the more female-centered thin ideal [41] and weight-gain tends to be associated with the attainment of the more male-centered muscular ideal [42], it is possible that men might view weight loss interventions as discordant with their health and fitness goals. This might be one of the reasons why male participants are substantially underrepresented in the literature on weight loss [43] and eating behaviors [44]. It might therefore seem appropriate to employ other strategies to attract men into studies that tackle obesity and eating pathology. Perhaps the use of a more “gender-neutral” emphasis like spirituality helped attract a more gender-balanced sample for this study.

High levels of depression were not surprising as it is often comorbid with obesity [45, 46] and eating disordered behavior [47, 48]. Previous studies also have found that high scores on the YFAS are associated with higher levels of depression [49, 50]. Being an ethnic minority having a lower income, and having a lower education are all risk factors for depression [51] and were relevant to our sample. Because this population is less likely to seek medical and mental health services, it is imperative that culturally congruent interventions continue to be developed which may attract individuals who would benefit not only from addressing weight-related issues but also form a gateway into the mental health care system. In our case, we believe that the emphasis on spirituality was the hook that brought them into a study that incorporated mental health treatment.

No specific issues that might suggest participant dissatisfaction arose during the course of the study. Despite elevated depression scores, none of the participants expressed distress that would have warranted higher levels of care, in our clinical estimation. Because of the reluctance of this population to seek out healthcare, we chose to house the intervention outside of a medical center. Distrust in health care professionals might be prevalent in this population, as mentioned earlier, but it is possible that the abundance of spiritual terminology inherent in the 3S protocol made the study team seem less like other health care professionals previously encountered.

The attrition rate in our study was low compared to other eating and weight interventions. Attrition this low (roughly 15.4%) is unusual in weight loss studies, even during the weight loss phase [52]. A meta-analysis examining weight loss initiatives specifically targeting African-American women reported that the attrition rate for most of those studies ranged from 23% to 47%, on average [53]. Attrition rates in weight loss studies tend to be higher among ethnic minorities [38, 54]. Discouragement over slow weight loss, dissatisfaction with prior weight loss efforts, and transportation issues have been cited as barriers to retaining African-Americans once enrolled in a study [55]. Participants in the current study were weighed at baseline and at study completion so it is possible that not focusing on weekly changes in body weight protected participants from an experience of shame, embarrassment, or dissatisfaction with weight loss and this, in turn, could have contributed to the higher levels of adherence.

Because the intervention was conducted in a predominantly African-American and Hispanic neighborhood, we expected to draw primarily ethnic minorities, even though there were no race- or ethnicity-based inclusion/exclusion criteria. Only two Caucasian participants were enrolled but they dropped out after sessions 4 and 6, respectively.

As described earlier, many participants expressed enthusiasm about getting others to enroll in the pilot study. This might therefore be an ideal project that can eventually be snowballed into a large-scale CBPR effort in which former participants can be elevated to investigators and help disseminate replications. We had a lot of difficulty garnering interest from community organizations but now that personal relationships have been developed with participants, they might be best suited to help broaden our relationships with the community.

Since this is a pilot study, our small sample size makes it difficult to draw conclusions about the population. However, our aim was to pilot-test this approach among a small sample in order to examine feasibility and acceptability. Another limitation of the study has to do with the compensation. It is unclear if participants would sign up for a weight loss study without monetary compensation. However, university-based weight loss programs typically do offer compensation [56] and they still do not tend to recruit minorities and men very easily [44, 57].

In conclusion, obese, inner-city minorities were attracted to an intervention that utilized their personal spirituality in conjunction with recognized techniques from cognitive therapy. Further studies of this dynamic are certainly warranted given the urgency of this health issue, lack of effective interventions, and needs of underserved minorities. There is a vital need to deliver effective, empirically based interventions to obese minorities [58, 59]. This pilot study showed feasibility in attracting a target population and was successful in retaining all of the participants who were identified as ethnic minorities. However, it is unclear if the current investigation will result in meaningful weight loss, especially as measured in the long-term. It is expected that continued development of spirituality-oriented interventions will readily engage participants that have historically been very difficult to reach and retain [15] despite demonstrated need for such services.

## Figures and Tables

**Table 1:** Spiritual Self-Schema Therapy (3S): outline of weekly sessions.

Session	Topic	Description
1	Introduction to 3S	The “Spiritual Self” and the Noble Eightfold Path

2	Training in Mastery of the Mind	1: Becoming Aware and Changing Habit Patterns of the Mind
3		2: Managing “Addict Self” Intrusions
4		3: Mindful Action versus Automatic Reaction

5	Training in Morality	1: Mindfulness of Basic Needs
6		2: Everyday Ethics
7		3: Preventing Harm to Self and Others

8	Training in Wisdom	1: Filling the Mind with the “Spiritual Self”
9		2: Coping with Stigma
10		3: Renouncing the “Addict Self” Identity and Fully Assuming the “Spiritual Self” Identity
11		4: Serenity and Insight

12	Termination and Transition	Maintaining the Spiritual Path

**Table 2:** Sample characteristics at baseline.

	*N*	*M*	SD
Age	13	42.46	9.54
BMI	13	36.18	4.75
CES-D	13	23.84	10.67
EDE-Q Global	13	3.28	1.54
BMMRS Daily Experience	13	4.71	1.16
BMMRS Coping	13	3.19	0.57
YFAS	13	4.00	2.31

## References

[1] Pi-Sunyer FX (1993). Medical hazards of obesity. *Annals of Internal Medicine*.

[2] Bruce SG, Riediger ND, Zacharias JM, Young TK (2011). Obesity and obesity-related comorbidities in a Canadian First Nation population. *Preventing Chronic Disease*.

[3] Jeffery RW, Epstein LH, Wilson GT (2000). Long-term maintenance of weight loss: current status. *Health Psychology*.

[4] Wing RR, Phelan S (2005). Long-term weight loss maintenance. *The American Journal of Clinical Nutrition*.

[5] Flegal KM, Carroll MD, Ogden CL, Curtin LR (2010). Prevalence and trends in obesity among US adults, 1999–2008. *Journal of the American Medical Association*.

[6] Carrington CH (2006). Clinical depression in African American women: diagnoses, treatment, and research. *Journal of Clinical Psychology*.

[7] Jupka KA, Weaver NL, Sanders-Thompson VL, Caito NM, Kreuter MW (2012). African American adults' experiences with the health care system: in their own words. *Journal of Health Disparities Research and Practice*.

[8] Neighbors HW, Caldwell C, Williams DR (2007). Race, ethnicity, and the use of services for mental disorders: results from the National Survey of American Life. *Archives of General Psychiatry*.

[9] Weinick RM, Zuvekas SH, Cohen JW (2000). Racial and ethnic differences in access to and use of health care services, 1977 to 1996. *Medical Care Research and Review*.

[10] Annunziato RA, Lowe MR (2007). Taking action to lose weight: toward an understanding of individual differences. *Eating Behaviors*.

[11] Armstrong K, McMurphy S, Dean LT (2008). Differences in the patterns of health care system distrust between blacks and whites. *Journal of General Internal Medicine*.

[12] Nicolaidis C, Tlmmons V, Thomas MJ (2010). ‘You don’t go tell white people nothing’: African American women’s perspectives on the influence of violence and race on depression and depression care. *American Journal of Public Health*.

[13] Powell LH, Shahabi L, Thoresen CE (2003). Religion and spirituality: linkages to physical health. *American Psychologist*.

[14] Gallup Poll. http://www.gallup.com/poll/1690/Religion.aspx%20.

[15] Stanley MA, Bush AL, Camp ME (2011). Older adults’ preferences for religion/spirituality in treatment for anxiety and depression. *Aging and Mental Health*.

[16] American Association of Pastoral Counselors Survey findings, November 8, 2000: American Association of Pastoral Counselors & The Samaritan Institute Report. http://aapc.org/content/public-opinion-poll.

[17] Snodgrass J (2009). Toward holistic care: integrating spirituality and cognitive behavioral therapy for older adults. *Journal of Religion, Spirituality and Aging*.

[18] Lapane KL, Lasater TM, Allan C, Carleton RA (1997). Religion and cardiovascular disease risk. *Journal of Religion and Health*.

[19] Kim KH, Sobal J, Wethington E (2003). Religion and body weight. *International Journal of Obesity*.

[20] Dodor B (2012). The impact of religiosity on health behaviors and obesity among African Americans. *Journal of Human Behavior in the Social Environment*.

[21] Avants SK, Margolin A (2004). Development of spiritual self-schema (3-S) therapy for the treatment of addictive and HIV risk behavior: a convergence of cognitive and Buddhist Psychology. *Journal of Psychotherapy Integration*.

[22] Marcotte D, Avants SK, Margolin A (2003). Spiritual Self-Schema therapy, drug abuse, and HIV. *Journal of Psychoactive Drugs*.

[23] Lipkus IM, Lyna PR, Rimer BK (1999). Using tailored interventions to enhance smoking cessation among African-Americans at a community health center. *Nicotine and Tobacco Research*.

[24] Longshore D, Grills C (2000). Motivating illegal drug use recovery: evidence for a culturally congruent intervention. *Journal of Black Psychology*.

[25] Gearhardt AN, Corbin WR, Brownell KD (2009). Preliminary validation of the Yale food addiction scale. *Appetite*.

[26] Fairburn CG, Beglin SJ (1994). Assessment of eating disorders: interview or self-report questionnaire?. *International Journal of Eating Disorders*.

[27] Cooper Z, Fairburn C (1987). The eating disorder examination: a semi-structured interview for the assessment of the specific psychopathology of eating disorders. *International Journal of Eating Disorders*.

[28] Cooper Z, Cooper PJ, Fairburn CG (1989). The validity of the eating disorder examination and its subscales. *British Journal of Psychiatry*.

[29] Aardoom JJ, Dingemans AE, Slof-Op’t Landt MC, Van Furth EF (2012). Norms and discriminative validity of the Eating Disorder Examination Questionnaire (EDE-Q). *Eating Behaviors*.

[30] Radloff LS (1977). The CES-D scale a self-report depression scale for research in the general population. *Applied Psychological Measurement*.

[31] Idler EL, Musick MA, Ellison CG (2003). Measuring multiple dimensions of religion and spirituality for health research: conceptual background and findings from the 1998 general social survey. *Research on Aging*.

[32] Johnstone B, McCormack G, Yoon DP, Smith ML (2012). Convergent/divergent validity of the brief multidimensional measure of religiousness/spirituality: empirical support for emotional connectedness as a “Spiritual” construct. *Journal of Religion and Health*.

[33] Bluthenthal RN, Jones L, Fackler-Lowrie N (2006). Witness for wellness: preliminary findings from a community-academic participatory research mental health initiative. *Ethnicity and Disease*.

[34] Daley CM, James AS, Ulrey E (2010). Using focus groups in community-based participatory research: challenges and resolutions. *Qualitative Health Research*.

[35] Freimuth VS, Quinn SC, Thomas SB, Cole G, Zook E, Duncan T (2001). African Americans’ views on research and the Tuskegee Syphilis study. *Social Science and Medicine*.

[36] Beglin SJ, Fairburn CG (1992). Evaluation of a new instrument for the detection of eating disorders in community samples. *Psychiatry Research*.

[37] Jacobson NS, Dobson KS, Truax PA (1996). A component analysis of cognitive-behavioral treatment for depression. *Journal of Consulting and Clinical Psychology*.

[38] Lowe MR, Tappe KA, Annunziato RA (2008). The effect of training in reduced energy density eating and food self-monitoring accuracy on weight loss maintenance. *Obesity*.

[39] Boltri JM, Davis-Smith YM, Zayas LE (2006). Developing a church-based diabetes prevention program with African Americans: focus group findings. *Diabetes Educator*.

[40] Kolotkin RL, Crosby RD, Williams GR (2002). Health-related quality of life varies among obese subgroups. *Obesity Research*.

[41] Thompson JK, Stice E (2001). Thin-ideal internalization: mounting evidence for a new risk factor for body-image disturbance and eating pathology. *Current Directions in Psychological Science*.

[42] Thompson J, Cafri GE

[43] Anderson JW, Konz EC, Frederich RC, Wood CL (2001). Long-term weight-loss maintenance: a meta-analysis of US studies. *American Journal of Clinical Nutrition*.

[44] Dakanalis A, Riva G (2013). Current considerations for eating and body-related disorders among men. *Handbook on Body Image: Gender Differences, Sociocultural Influences and Health Implications*.

[45] Onyike CU, Crum RM, Lee HB, Lyketsos CG, Eaton WW (2003). Is obesity associated with major depression? Results from the third National Health and Nutrition Examination survey. *American Journal of Epidemiology*.

[46] Stunkard AJ, Faith MS, Allison KC (2003). Depression and obesity. *Biological Psychiatry*.

[47] Braun DL, Sunday SR, Halmi KA (1994). Psychiatric comorbidity in patients with eating disorders. *Psychological Medicine*.

[48] Casper RC (1998). Depression and eating disorders. *Depression and Anxiety*.

[49] Burmeister JM, Hinman N, Koball A, Hoffmann DA, Carels RA (2013). Food addiction in adults seeking weight loss treatment. Implications for psychosocial health and weight loss. *Appetite*.

[50] Davis C, Carter JC (2009). Compulsive overeating as an addiction disorder. A review of theory and evidence. *Appetite*.

[51] Hudson DL, Neighbors HW, Geronimus AT, Jackson JS (2012). The relationship between socioeconomic position and depression among a US nationally representative sample of African Americans. *Social Psychiatry and Psychiatric Epidemiology*.

[52] Wadden TA, Foster GD, Letizia KA, Stunkard AJ (1992). A multicenter evaluation of a proprietary weight reduction program for the treatment of marked obesity. *Archives of Internal Medicine*.

[53] Bronner Y, Boyington JEA (2002). Developing weight loss interventions for African-American women: elements of successful models. *Journal of the National Medical Association*.

[54] Jelalian E, Hart CN, Mehlenbeck RS (2008). Predictors of attrition and weight loss in an adolescent weight control program. *Obesity*.

[55] Pleas J (1988). Long-term effects of a lifestyle-change obesity treatment program with minorities. *Journal of the National Medical Association*.

[56] Paul-Ebhohimhen V, Avenell A (2008). Systematic review of the use of financial incentives in treatments for obesity and overweight. *Obesity Reviews*.

[57] Tsai AG, Wadden TA, Pillitteri JL (2009). Disparities by ethnicity and socioeconomic status in the use of weight loss treatments. *Journal of the National Medical Association*.

[58] Paskett ED, Reeves KW, McLaughlin JM (2008). Recruitment of minority and underserved populations in the United States: the centers for population health and health disparities experience. *Contemporary Clinical Trials*.

[59] Yancey AK, Ortega AN, Kumanyika SK (2006). Effective recruitment and retention of minority research participants. *Annual Review of Public Health*.

